# Implementation of Suicide Risk Estimation Analytics to Support Mental Health Care for Quality Improvement

**DOI:** 10.1001/jamanetworkopen.2022.47195

**Published:** 2022-12-16

**Authors:** Julie E. Richards, Bobbi Jo H. Yarborough, Erika Holden, Lisa Shulman, Scott P. Stumbo, Yates Coley, Gregory E. Simon

**Affiliations:** 1 Kaiser Permanente Washington Heath Research Institute, Seattle; 2 Department of Health Systems & Population Health, University of Washington, Seattle; 3 Kaiser Permanente Northwest Center for Health Research, Portland, Oregon; 4 Department of Biostatistics, University of Washington, Seattle; 5 Psychiatry and Behavioral Sciences, University of Washington, Seattle; 6 Kaiser Permanente Washington Department of Mental Health & Wellness, Seattle, Washington

## Abstract

This quality improvement study describes use of estimation analytics to augment existing suicide prevention practices during routine mental health specialty encounters at a large US health care system.

## Introduction

Suicide risk estimation analytics combine use of sociodemographic and clinical characteristics to quantify risk for defined populations. Development and testing have been promising,^1,2^ but there is very little evidence to guide routine use of estimation models during clinical encounters. The most well-known efforts have focused on outreach*,* such as ReachVet which successfully implemented suicide risk estimation analytics to identify at-risk veterans and address care needs.^3^ Encounter-based implementation efforts have not been described. Therefore, this mixed-methods quality improvement study describes use of estimation analytics to augment existing suicide prevention practices during routine mental health specialty encounters at Kaiser Permanente Washington.

## Methods

The Kaiser Permanente institutional review board approved a waiver of consent and HIPAA authorization for access, use, and collection of protected health information from medical records to conduct this study, which followed the SQUIRE quality improvement reporting guideline. Preexisting clinical workflows included administration of the Patient Health Questionnaire-9 (PHQ-9) prior to all encounters with patients (aged at least 13 years), and an electronic health record (EHR) prompt to complete the Columbia-Suicide Severity Rating Scale (C-SSRS) among patients reporting frequent thoughts about self-harm (score 2 to 3 on PHQ-9 question 9). A previously developed and validated prediction model^1,2^ was implemented via a schedule-based flag to prompt additional C-SSRS administration among patients identified at high risk of suicide attempt (at least 3% in the following 90 days), regardless of their PHQ-9 ninth question responses (see eMethods in Supplement 1 for additional detail). Clinicians received directions to display the schedule-based flag and scripting to help normalize the new risk assessment workflow for patients.

EHR data was obtained for a postimplementation observation period (December 1, 2019, to March 15, 2020) to assess how often the C-SSRS was completed among flagged encounters. In parallel, a sample of flagged 30 adult patients (March 13, 2020, to September 17, 2020) and all 16 clinicians (May 11, 2020, to June 22, 2020) were invited to participate in semistructured audio-recorded virtual interviews (as part of a broader multisite qualitative study),^4^ locally analyzed using thematic triangulation via Atlas.ti software version 9 (Scientific Software Development GmbH).

## Results

There were 4789 patient encounters (3102 [65%] female; 342 [7%] Asian; 286 [6%] Black; 398 [8%] Hawaiian or Pacific Islander; 3350 [70%] White; 2681 [56%] aged 18 to 39 years) by 1939 patients in the 3.5-month observation period, including 161 (3.5%) encounters newly flagged via suicide risk estimation analytics (Table).

**Table.** Patient Encounter Characteristics During the Study Period, December 1, 2019, to March 15, 2020

Characteristic	Flag, No. (%)		*P* value
	No (n = 4628)	Yes (n = 161)	
Age, y			
13-17	247 (5)	19 (12)	<.001
18-39	2563 (55)	118 (73)	
40-64	1294 (28)	18 (11)	
≥65	524 (11)	6 (4)	
Sex			
Male	1639 (35)	48 (30)	.14
Female	2989 (65)	113 (70)	
Race			
American Indian or Alaska Native	67 (1)	7 (4)	<.001
Asian	339 (7)	3 (2)	
Black	285 (6)	1 (1)	
Hawaiian or Pacific Islander	387 (8)	11 (7)	
Hispanic or Latino/a/x	48 (1)	1 (1)	
Other^a^	114 (2)	10 (6)	
Unknown	159 (3)	7 (4)	
White	3229 (70)	121 (75)	
Insurance type			
Not insured	361 (8)	0 (0)	<.001
Commercial	2766 (60)	115 (71)	
Medicaid	220 (5)	0 (0)	
Medicare	716 (15)	20 (12)	
Private pay	565 (12)	26 (16)	
Depression severity^b^			
Missing	793 (17)	30 (19)	<.001
None-minimal	867 (19)	2 (1)	
Mild	1114 (24)	24 (15)	
Moderate	907 (20)	27 (17)	
Moderate-severe	549 (12)	35 (22)	
Severe	398 (9)	43 (27)	
Frequency of suicidal ideation^c^			
Missing	805 (17)	29 (18)^d^	<.001
Not at all (score = 0)	2684 (58)	25 (16)^d^	
Several days (score = 1)	734 (16)	50 (31)^d^	
More than half the days (score = 2)	234 (5)	30 (19)	
Nearly every day (score = 3)	171 (4)	27 (17)	
Mental health diagnoses (prior year)			
Depression	3282 (71)	138 (86)	<.001
Anxiety	3928 (85)	161 (100)	<.001
Serious mental illness^e^	882 (19)	84 (52)	<.001
Suicide attempt	42 (1)	26 (16)	<.001

^a^ Other race and ethnicity was used to broadly capture all other write-in responses that do not fall into one of the broader categories, such as “Irish,” “Ashkenazi Jewish,” or “human race.”

^b^ The Patient Health Questionnaire (PHQ-9) was used to measure depressive symptom severity.

^c^ Measured via PHQ-9 Q9: “Thoughts that you would be better off dead, or of hurting yourself (prior 2 weeks).”

^d^ Newly flagged encounters referenced in Results.

^e^ Serious mental illness diagnoses include bipolar, schizophrenia, other psychosis or personality disorders. The diagnostic codes used to create the analytic data set used for this analysis are publicly available from the Mental Health Research Network (eMethods in Supplement 1).

The encounter-based risk flag did not consistently prompt additional suicide risk assessment as intended. During newly flagged encounters: 57 patients reported frequent suicidal ideation (PHQ-9 question 9 [Q9] ≥ 2) and, as per preexisting workflow, 54 (95%) completed a C-SSRS; 75 patients reported no or infrequent suicidal ideation (Q9 = 0 to 1), but only 10 (13%) completed a C-SSRS, per new workflow; 29 patients did not answer the PHQ-9, and only 1 patient (3%) completed a C-SSRS, per new workflow.

Interviewed clinicians (n = 8) identified important implementation concerns, including (1) lack of follow-up, (2) EHR-related inefficiencies, and (3) reliability and accuracy of the flag. Interviewed patients (n = 20) reiterated concerns about reliability and accuracy of estimation analytics. Additionally, clinicians described concerns about access to care and potential liability associated with known suicide risk. Patients echoed concerns about access and expressed fears about identification of suicide risk resulting in coercive care (Figure).

**Figure.**
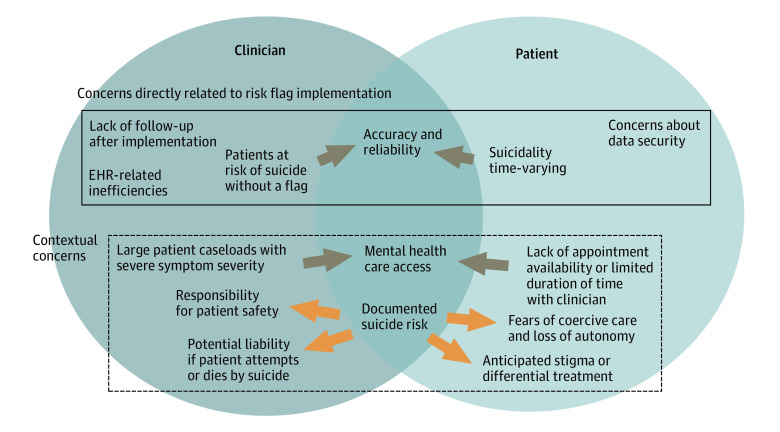
Clinician (N = 8) and Patient (N = 20) Concerns About Implementation of Suicide Risk Estimation Analytics: Triangulation of Semistructured Interview Themes Dark arrows indicate convergence between patient and clinician interviews; light arrows indicate divergence.

## Discussion

This novel quality improvement study highlights important implications for health care organizations considering implementation of estimation analytics to support encounter-based identification of suicide risk. First, while the schedule-based flag was simple and inexpensive to implement, it was not effective—a user-centered approach to clinical decision support design is key for prompting intended actions. Second, bidirectional leadership/clinician communication is critical for addressing implementation outcomes and concerns. For example, opportunities to underscore that suicide risk estimation analytics are designed to augment rather than replace clinical judgement and screening or assessment practices may mitigate concerns about reliability and accuracy. Limitations of this study included timing–redesign efforts will support virtual mental health care delivery (commonplace post-COVID). Finally, findings underscore tensions between clinician concerns about responsibility for patient safety and patient concerns about coercive care.^5^ Therefore, use of estimation analytics and other suicide prevention tools and practices should not reinforce a “culture of blame,”^6^ but rather support therapeutic alliance.

## Notes

Supplement 1. **eMethods.** Supplemental Methods

Supplement 2.
Data Sharing Statement
