# Supplementary material for: Implementation of Suicide Risk Estimation Analytics to Support Mental Health Care for Quality Improvement
Source: JAMA Netw Open. 2022 Dec 16;5(12):e2247195. doi: 10.1001/jamanetworkopen.2022.47195 (PMC9856428; doi:10.1001/jamanetworkopen.2022.47195)
Supplement: Supplement 1. — eMethods. Supplemental Methods [file jamanetwopen-e2247195-s001.pdf]

Supplemental Online Content

Richards JE, Yarborough BJH, Holden E, et al. Implementation of suicide risk estimation analytics to support mental health care for quality improvement. *JAMA Netw Open.* 2022;5(12):e2247195. doi:10.1001/jamanetworkopen.2022.47195

eMethods. Supplemental Methods

This supplemental material has been provided by the authors to give readers additional information about their work.

© 2022 Richards JE et al. *JAMA Network Open.*

eMethods. Supplemental Methods

Study Design

This mixed-method (QUAN + qual)[1, 2] quality improvement evaluation examined the implementation of suicide risk predictive analytics during routine mental health specialty outpatient encounters. Specifically, descriptive statistical analyses were used to examine whether the implementation of the scheduled-based risk flag worked as intended, and qualitative analyses were used to explore patient and clinician experiences with the implementation and perceptions of suicide risk predictive analytics.

Healthcare System Setting & Data Sources

Kaiser Permanente Washington (KPWA) provides comprehensive medical and psychiatric specialty care to a defined population of about 700,000 members who are enrolled through employer-sponsored or individual insurance plans or capitated Medicaid or Medicare programs. KPWA maintains a robust electronic health record database that captures members' demographic, enrollment, and clinical/diagnostic information, prescription dispensing data, internal service utilization, external health care claims data, and mortality data. Electronic health records data are organized into a research virtual data warehouse, using standard definitions across the Health care Systems Research Network and are quality checked locally.[3]

Suicide Risk Prediction Algorithm Implementation

Implementation was designed to augment the existing clinical workflow, which involves administration of the Patient Health Questionnaire-9 (PHQ-9) [4] prior to all encounters with patients (age 13+), followed by the Columbia-Suicide Severity Rating Scale (C-SSRS) [5] among patients reporting frequent thoughts about self-harm (score 2–3 on PHQ-9 question 9), followed by safety planning[6] with patients reporting any prior month intent or planning for suicide. A new schedule-based flag (Figure) used a previously developed and validated model designed to predict suicide risk in the 90 days following a mental health specialty encounter.[7-9] Suicide risk flag implementation was designed to use minimal resources (i.e., clinical decision support programming). Clinicians received information about the flag at a team meeting on 10/8/2019, in addition to a "huddlecard" (see Text Box below) with additional information about how to introduce the C-SSRS to patients and directions to add the flag to their schedules. There were no additional implementation activities (e.g. communications, trainings, performance feedback), due in part to the COVID-19 pandemic and care delivery disruptions.

Figure: Screenshot of scheduled-based flag to prompt suicide risk assessment (C-SSRS).

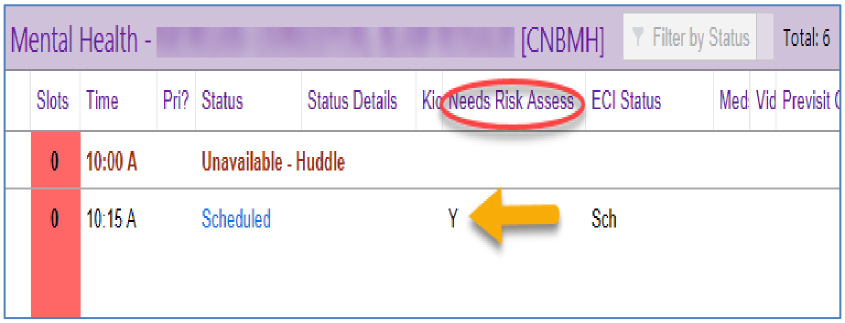

Descriptive Statistical Analyses: Sample & Dataset Specifications

Below is a description of patient sample and approach used to assemble the encounter-level dataset for the descriptive statistical analyses presented in this evaluation.

<u>Sample:</u> All in-person or virtual encounters 12/1/19-3/15/2020 (approximately one month after implementation until COVID-19 related care disruptions) to the outpatient mental health specialty clinic selected by health system leaders to implement suicide risk predictive analytics. Patients younger than 13 were excluded, which is the age at which patients can consent to receive mental health care in Washington State and the risk prediction algorithm had been validated.[8]

The study programmer analyst followed this approach to create the analytic dataset:

© 2022 Richards JE et al. *JAMA Network Open.*

Step 1: identified encounters usingdepartment specialty information to limit to encounters within the implementation clinic, created a visit order variable and indicator for the first visit in the dataset, and linked PHQ-9 and C-SSRS data documented in the medical record at the time of each patient encounter (if done, missing if not done).
Step 2: Added a suicide risk flag to each encounter, using the dataset used to populate the scheduled-based suicide risk flag (via electronic health record datalink process).
Step 3: Linked patient-level VDW demographic data to encounters, including age, sex, race & ethnicity, insurance type, and prior-year diagnosis data (details available here: https://github.com/MHResearchNetwork/Diagnosis-Codes)

Descriptive statistical analyses compared sociodemographic and clinical characteristics of patient encounters with and without a suicide risk flag following implementation.

Qualitative Analyses
Sample & Approach: Qualitative data collection was done in collaboration with Kaiser Permanente Northwest and HealthPartners in Minnesota, as part of a broader multi-site research project.[10] Qualitative analysis for purposes of this quality improvement study was done separately by a local team of qualitative researchers at Kaiser Permanente Washington. Below we describe the sampling and recruitment procedures and approach to qualitative analysis.

Patient Sampling & Recruitment: Adult patients (age ≥18) who had been flagged at high risk of suicide attempt to prompt C-SSRS administration were identified and sampled using electronic health record data during the interview period. A study team member pulled lists of eligible patients using EHR data and assigned a unique study ID to each sampled patient and used a Research Electronic Data Capture (REDCap) tracking database, accessible only to the KPWA study interviewers, that included patient names and contact information. As participants were recruited for interview participation, the project leader and adjusted sampling targets to increase demographic diversity (gender, age, race and ethnicity). Patients were mailed invitations, including an information sheet with instructions for opting out. Interviewers attempted to call invited patients within two weeks of invitation. Participants provided oral consent and received $50 cash. At the time of the interview, patients were not aware they had been identified as at higher risk of suicide attempt, but were provided general information about suicide risk prediction algorithms (e.g. purpose, types of data used) to elicit their feedback. Recruitment continued in waves until the recruitment target (N=20) was reached.

Clinician Recruitment: All mental health clinicians within the implementation clinic received up to three email invitations for interview participation, including an information sheet with instructions for opting out. Participants provided oral consent and received an $50 e-gift card.

Telephone Interviews: Three interviewers, including one doctoral-level public health researcher (JR) and one masters-level social worker (LS), and one experienced interviewer with an undergraduate-level background in anthropology (EH) conducted semi-structured phone interviews (~30 minutes long). As previously described,[10, 11] interview guides were informed by prior qualitative study themes[12, 13] and designed in consultation with researchers, clinicians, and individuals who had received mental health care. Interviews were audio-recorded and professionally transcribed.

Data Analysis: Study interviewers coded transcripts in Atlas.ti using both directive (deductive) and conventional (inductive) content analysis.[14] Two staff independently coded each transcript with iterative comparison/discrepancy resolution during weekly meetings. Clinician interviews were analyzed first, and codes were organized into thematic networks[15] to facilitate discussions. Patient interviews were then coded and triangulation methods used to support analysis of *convergence*, *complementarity*, and

divergence between interview respondent groups.[16] Findings were presented to the research team and clinical stakeholders for further review and refinement.

Text Box: Excerpt from the implementation huddlecard

Huddle Card – New Suicide Risk Flag in HealthConnect

WHY? We now use Item 9 of the PHQ9 (about "Thoughts you would be better off dead, or of hurting yourself") to identify people at increased risk for suicide attempt. This simple method is reasonably accurate, but it misses over half of people who later attempt or die by suicide. Suicidal ideation may come and go over time, and some people may not want to reveal suicidal thoughts. We can identify risk more accurately if we consider other information in our records (prior suicide attempts, hospitalizations, ER/urgent care visits, specific diagnoses). Using information from records can also identify risk in people who do not complete the PHQ9. And we can identify risk in advance, even before a patient arrives.

WHAT IS CHANGING? Starting November 7th, you will see a new column labelled "Needs Risk Assessment" in your Epic schedule. A "Y" in that column means that person has at least a 3% risk of suicide attempt in the following 90 days, sometimes higher. That risk is high enough that we should assess further – using our standard Columbia risk assessment – regardless of the response to Item 9 of the PHQ9. Most of the people identified by high risk scores are the same people we already identify using the PHQ9. But some are high-risk people we have been missing.

HOW? When you see "Y" in the "Needs SRA" column, you'll know that your visit should include a risk assessment using the Columbia scale. In most cases, people a "Needs SRA" flag will also report frequent suicidal ideation on Item 9 of the PHQ9. In those cases, you would just follow our existing standard work:

- Complete or update a risk assessment using the Columbia scale
- Create or update a crisis response plan (aka safety plan) depending on the Columbia score, using the standardized template .SUICIDECRISISRESPONSEPLAN

In some cases, a person with a "Needs SRA" flag will score low on item 9 of the PHQ9 – or not complete the PHQ9 at all. That's when your work will change – completing a Columbia risk assessment when you might not have before**. If the score on PHQ9 item 9 is 0 or 1, the Columbia flow sheet will not pop up automatically.** But you can find it by clicking on "Flowsheet" on the right side of the "Vitals" section under the "Rooming Tab." Adding the Columbia to your list of favorite flowsheets will make this step easier.

Example Scripting for introducing the SRA to patients with a missing PHQ-9 or ninth question score 0 or 1:

- If the PHQ9 was not completed: "I see you weren't able to complete our progress monitoring questionnaire. So I'd like to ask you a few specific questions that we always want to check on."
- If the response to PHQ9 item 9 was "Not at all" (score 0): "I see that you said you don't have thoughts you'd be better off dead or thoughts of hurting yourself. I'd still like to ask you a few follow-up questions about that."
- If the response to PHQ9 item 9 was "Several Days" (score 1): "I see that you sometimes do have thoughts you would be better off dead or thoughts about hurting yourself. I'd like to ask you some follow-up questions about that."

© 2022 Richards JE et al. *JAMA Network Open.*

References

1. Creswell JW, Clark VLP. *Designing and conducting mixed methods research*. Sage publications; 2017.

2. Teddlie C, Tashakkori A. Major issues and controversies in the use of mixed methods in the social and behavioral sciences. *Handbook of mixed methods in social and behavioral research*. 2003;1(1):13-50.

3. The Health Care Systems Research Network. Data Resources. Kaiser Permanente Division of Research (Northern California). Accessed August 19, 2021. http://www.hcsrn.org/en/About/Data/

4. Kroenke K, Spitzer RL, Williams JB. The PHQ-9: validity of a brief depression severity measure. *J Gen Intern Med*. Sep 2001;16(9):606-13. doi:jgi01114 [pii]

5. Posner K, Brown GK, Stanley B, et al. The Columbia-Suicide Severity Rating Scale: initial validity and internal consistency findings from three multisite studies with adolescents and adults. *Am J Psychiatry*. Dec 2011;168(12):1266-77. doi:10.1176/appi.ajp.2011.10111704

6. Stanley B, Brown GK. Safety planning intervention: a brief intervention to mitigate suicide risk. *Cogn Behav Pract*. 2012;19(2):256-264.

7. Simon GE, Johnson E, Lawrence JM, et al. Predicting Suicide Attempts and Suicide Deaths Following Outpatient Visits Using Electronic Health Records. *Am J Psychiatry*. Oct 1 2018;175(10):951-960. doi:10.1176/appi.ajp.2018.17101167

8. Penfold RB, Johnson E, Shortreed SM, et al. Predicting suicide attempts and suicide deaths among adolescents following outpatient visits. *J Affect Disord*. Nov 1 2021;294:39-47. doi:S0165-0327(21)00643-1 [pii]

10.1016/j.jad.2021.06.057

9. Cruz M, Shortreed SM, Richards JE, et al. Machine Learning Prediction of Suicide Risk Does Not Identify Patients Without Traditional Risk Factors. *J Clin Psychiatry*. Aug 31 2022;83(5)doi:10.4088/JCP.21m14178

21m14178 [pii]

10. Yarborough BJH, Stumbo SP, Schneider JL, Richards JE, Hooker SA, Rossom RC. Patient expectations of and experiences with a suicide risk identification algorithm in clinical practice. *BMC Psychiatry*. Jul 23 2022;22(1):494. doi:10.1186/s12888-022-04129-1

10.1186/s12888-022-04129-1 [pii]

11. Yarborough BJ, Stumbo SP, Schneider JL, Richards JE, Hooker SA, Rossom RC. Clinical Implementation of Suicide Risk Prediction Models in Healthcare. Under review 2022.

12. Yarborough BJH, Stumbo SP. A Stakeholder-Informed Ethical Framework to Guide Implementation of Suicide Risk Prediction Models Derived from Electronic Health Records. *Arch Suicide Res*. Apr 21 2022:1-14. doi:10.1080/13811118.2022.2064255

13. Yarborough BJH, Stumbo SP. Patient perspectives on acceptability of, and implementation preferences for, use of electronic health records and machine learning to identify suicide risk. *Gen Hosp Psychiatry*. May-Jun 2021;70:31-37. doi:S0163-8343(21)00028-1 [pii]

10.1016/j.genhosppsych.2021.02.008

14. Hsieh HF, Shannon SE. Three approaches to qualitative content analysis. *Qual Health Res*. Nov 2005;15(9):1277-88. doi:15/9/1277 [pii]

10.1177/1049732305276687

15. Attride-Stirling J. Thematic networks: an analytic tool for qualitative research. *Qualitative research*. 2001;1(3):385-405.

16. Farmer T, Robinson K, Elliott SJ, Eyles J. Developing and implementing a triangulation protocol for qualitative health research. *Qual Health Res*. Mar 2006;16(3):377-94. doi:10.1177/1049732305285708

© 2022 Richards JE et al. *JAMA Network Open.*
