# Supplementary material for: Implementation of Suicide Risk Estimation Analytics to Support Mental Health Care for Quality Improvement
Source: JAMA Netw Open. 2022 Dec 16;5(12):e2247195. doi: 10.1001/jamanetworkopen.2022.47195 (PMC9856428; doi:10.1001/jamanetworkopen.2022.47195)
Supplement: Supplement 2. — Data Sharing Statement [file jamanetwopen-e2247195-s002.pdf]

Data Sharing Statement

Richards. Implementation of Suicide Risk Estimation Analytics to Support Mental Health Care for Quality Improvement Evaluation. *JAMA Netw Open*. Published December 16, 2022. doi:10.1001/jamanetworkopen.2022.47195

Data
Data available: No

Additional Information
Explanation for why data not available: In keeping with safeguards required by the Health Insurance Portability and Accountability (HIPAA) Privacy and Security Rules we will not make all datasets publicly available due to risk of re-identification and breach of patient confidentiality. Qualitative interview guides are available to interested researchers upon request. The diagnostic codes used to create the analytic dataset used for this analysis are publicly available from the Mental Health Research Network: https://github.com/MHResearchNetwork/Diagnosis-Codes.
